# Carboxylesterase Activities and Protein Expression in Rabbit and Pig Ocular Tissues

**DOI:** 10.1021/acs.molpharmaceut.0c01154

**Published:** 2021-02-17

**Authors:** Anam Hammid, John K. Fallon, Toni Lassila, Giulia Salluce, Philip C. Smith, Ari Tolonen, Achim Sauer, Arto Urtti, Paavo Honkakoski

**Affiliations:** † School of Pharmacy, University of Eastern Finland, Yliopistonranta 1 C, 70210 Kuopio, Finland; ‡ Division of Pharmacoengineering and Molecular Pharmaceutics, Eshelman School of Pharmacy, University of North Carolina at Chapel Hill, Campus Box 7355, Chapel Hill, North Carolina 27599-7355, United States; § Admescope Ltd, Typpitie 1, 90620 Oulu, Finland; ∥ Centro Singular de Investigación en Química Biolóxica e Materiais Moleculares (CiQUS), Departamento de Química Orgánica, Universidade de Santiago de Compostela, 15782 Santiago de Compostela, Spain; ⊥ Department of Drug Discovery Sciences, Boehringer Ingelheim Pharma GmbH & Co. KG, 88397 Biberach, Germany; # Institute of Chemistry, Saint Petersburg State University, Universitetskii pr. 26, 198584 Saint Petersburg, Russia; ∇ Faculty of Pharmacy, University of Helsinki, Viikinkaari 5 E, 00790 Helsinki, Finland; ○ Division of Pharmacotherapy and Experimental Therapeutics, Eshelman School of Pharmacy, University of North Carolina at Chapel Hill, Campus Box 7569, Chapel Hill, North Carolina 27599-7569, United States

**Keywords:** Ocular tissues, carboxylesterase, arylacetamide deacetylase, pig, rabbit, targeted proteomics

## Abstract

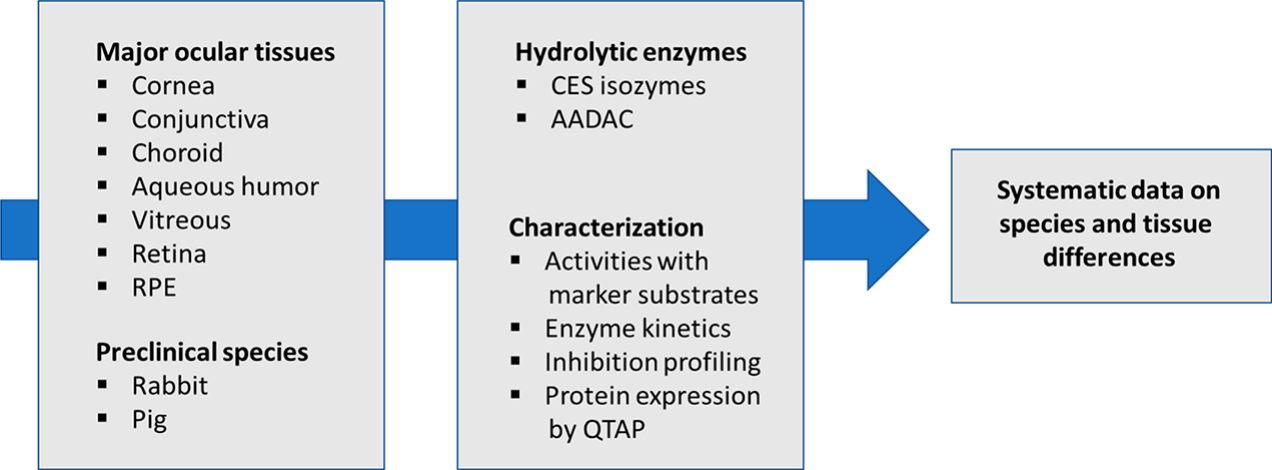

Hydrolytic reactions constitute an important pathway of drug metabolism and a significant route of prodrug activation. Many ophthalmic drugs and prodrugs contain ester groups that greatly enhance their permeation across several hydrophobic barriers in the eye before the drugs are either metabolized or released, respectively, *via* hydrolysis. Thus, the development of ophthalmic drug therapy requires the thorough profiling of substrate specificities, activities, and expression levels of ocular esterases. However, such information is scant in the literature, especially for preclinical species often used in ophthalmology such as rabbits and pigs. Therefore, our aim was to generate systematic information on the activity and expression of carboxylesterases (CESs) and arylacetamide deacetylase (AADAC) in seven ocular tissue homogenates from these two species. The hydrolytic activities were measured using a generic esterase substrate (4-nitrophenyl acetate) and, in the absence of validated substrates for rabbit and pig enzymes, with selective substrates established for human CES1, CES2, and AADAC (d-luciferin methyl ester, fluorescein diacetate, procaine, and phenacetin). Kinetics and inhibition studies were conducted using these substrates and, again due to a lack of validated rabbit and pig CES inhibitors, with known inhibitors for the human enzymes. Protein expression levels were measured using quantitative targeted proteomics. Rabbit ocular tissues showed significant variability in the expression of CES1 (higher in cornea, lower in conjunctiva) and CES2 (higher in conjunctiva, lower in cornea) and a poor correlation of CES expression with hydrolytic activities. In contrast, pig tissues appear to express only CES1, and CES3 and AADAC seem to be either low or absent, respectively, in both species. The current study revealed remarkable species and tissue differences in ocular hydrolytic enzymes that can be taken into account in the design of esterase-dependent prodrugs and drug conjugates, the evaluation of ocular effects of systemic drugs, and in translational and toxicity studies.

## Introduction

Hydrolysis plays a vital role in the metabolism or activation of many clinical (pro)drugs, such as cancer therapeutics, opioids, and angiotensin-converting enzyme inhibitors, as well as in lipid hydrolysis and the elimination of potentially toxic xenobiotics.^1−3^ Among many hydrolytic enzymes, carboxylesterases (CES, E.C. 3.1.1.1) are a group of serine hydrolases that cleave esters, amides, thioesters, and carbamates in numerous tissues including liver, kidney, intestine, placenta, heart, brain, and eye.^4−8^ So far, six human, about 20 rodent [including eight Ces1 (Ces1a–h), eight Ces2 (Ces2a–h), two Ces3 (Ces3a–b), and one each of Ces4 and Ces5 isoforms],^9,10^ three pig, and four rabbit CES proteins have been annotated in the Uniprot database (https://www.uniprot.org/uniprot/?query=CES&sort=score). Among the five functional human CES enzymes, CES1, and CES2 isoforms are the best-studied and major drug-metabolizing enzymes.^11−13^ In addition, CES1 is a major lipid-hydrolyzing enzyme.^3^ CES1 and CES2 share moderate sequence homology (47%) and display unique substrate specificities (Table 1).^2,11^ In a nutshell, substrates with a large acyl group and a small alcohol group are preferably metabolized by CES1. In contrast, CES2 prefers substrates with a small acyl and a large alcohol moiety.

**Table 1:** Examples of Drugs Metabolized by CES and AADAC Enzymes

enzyme	drug substrates	selective probe substrate	references
CES1	imidapril, enalapril, meperidine, oseltamivir, clopidogrel, heroin, lidocaine, irinotecan, cocaine	d-luciferin methyl ester (DME)	(12−16)
CES2	prasugrel, irinotecan, capecitabine	fluorescein diacetate (FDA), procaine	(14, 16, and 17)
AADAC	rifabutin, rifampicin, indiplon, rifapentine, flutamide	phenacetin	(16 and 18−21)

CES1 and CES2 are widely expressed in various tissues of multiple species. CES1 is the major hepatic CES isoform but is also present in the lungs, heart, and adipose tissue, while CES2 is quite abundant in the intestine but also expressed in the kidney, liver, adrenal, and stomach.^9^ The poorly studied CES3 isoform is expressed in the colon, brain, and at lower levels in the liver. It has much lower activity than CES1 or CES2 but may participate in irinotecan hydrolysis and in lipolysis in adipocytes.^16,19,22−24^ The metabolic roles of CES4 and CES5 are unknown, but they are expressed in neuronal cells.^25−27^ CES6 is present in human and mouse cerebellums and is speculated to take part in detoxification within the neural system.^28^ Arylacetamide deacetylase (AADAC)^16^ is another serine hydrolase and has recently emerged as an important drug-metabolizing enzyme with a substrate specificity overlapping with CESs. It is known to prefer substrates with the smallest acyl moieties (Table 1).^29^ The expression of human, rat, mouse, cynomolgus monkey, and dog AADAC has been found in the liver, kidney, and intestine.^27,29^

The eye is a complex organ composed of multiple cell types in the anterior (conjunctiva, cornea, iris ciliary body, aqueous humor, and lens) and posterior segments (vitreous humor, retina, retinal pigment epithelium (RPE), choroid, and sclera).^30^ However, their capacity for drug metabolism is not very well understood.^31^ The ocular hydrolysis of ester prodrugs has been reported in various species. Examples include the conversion of dipivefrin to epinephrine^32,33^ and the hydrolysis of latanoprost^34^ in rabbit corneal homogenates, and increases in the ocular bioavailability of pilocarpine,^35^ timolol,^36^ and ganciclovir by utilizing their ester prodrugs^37,38^ in corneal and choroidal tissues. Likewise, the cleavage of the carboxylic ester moiety of loteprednol etabonate in rabbit cornea, aqueous humor, and iris–ciliary body highlights the presence and activity of esterases in these tissues.^39^ The hydrolysis of nonspecific esterase substrates such as 4-nitrophenyl acetate (NPA) and naphthyl acetate has been observed in multiple rabbit and bovine ocular tissue homogenates.^8,22,32,37,40,41^ However, the enzymes responsible for these activities have not been characterized beyond the determination of kinetic parameters in a few cases.^32^ A review on global proteomics studies^42^ indicated that CES1 is detectable in most human ocular tissues except aqueous humor but quantitative expression data is nonexistent, and information on CES expression in other species is lacking, hampering translational studies. Finally, there is no knowledge on the ocular expression and activity of AADAC in the literature.^43^ To understand the biotransformation capacity in the eye and the risk of the formation of toxic metabolites from extended drug exposure from ocular implants, the metabolic profiling of individual ocular tissues in all preclinical species and in humans is crucial. A more detailed characterization of the catalytic and expression properties of ocular esterases is also important in the design of ophthalmic (pro)drugs and drug conjugates and in understanding the hydrolysis of drugs entering the eye from the systemic circulation.

Therefore, we aimed to characterize the activities and protein expression of CES1, CES2, and CES3 isoforms and AADAC that are metabolically active among mammalian species. Because of the unavailability of recombinant CES enzymes and the lack of information on their substrate selectivity in rabbits and pigs, multiple substrates characterized for the corresponding human CES enzymes were used. Kinetic and inhibition studies in select tissues with the highest activities were conducted. Finally, we performed a quantitative targeted proteomic analysis of the tissues to provide a systematic analysis of ocular hydrolytic enzymes in these two common preclinical species.

## Materials and Methods

### Chemicals and Reagents for Enzymatic Assays

4-Nitrophenyl acetate (NPA), 4-nitrophenol, acetonitrile (ACN), fluorescein diacetate (FDA), d-luciferin, Bio-Rad protein assay dye reagent (# 500-0006), diltiazem hydrochloride (purity ≥99%), timolol maleate salt (≥98%), verapamil hydrochloride (≥99%), fluorescein, bovine serum albumin (BSA), phenylmethylsulfonyl fluoride (PMSF), protease inhibitor cocktail (#P8340), phenacetin, *p*-phenetidine (≥99.9%), acetaminophen, procainamide-HCl (≥98%), procaine-HCl (>97%), *p-*aminobenzoic acid (PABA), potassium phosphate buffer (PBS), and dimethyl sulfoxide (DMSO; ≥99.9%) were purchased from Sigma-Aldrich Finland Oy (Espoo, Finland). Dulbecco’s phosphate-buffered saline (10 × DPBS; # 14200166) and phosphate-buffered saline (PBS) were obtained from Thermo Fisher. Luciferin methyl ester (DME) was purchased from the AAT bioquest (Sunnyvale, CA). QuantiLum recombinant luciferase (rLuciferase) was from Promega (Madison, WI). Digitonin (≥99%) was purchased from Merck Life Science (Merck KGaA, Darmstadt, Germany). All other chemicals were at least analytical grade.

### Chemicals and Reagents for Targeted Proteomics

Synthetic stable isotope-labeled (SIL) peptides (SpikeTides_TQL) that are isotopically labeled and quantified (Table 2) were obtained from JPT Peptide Technologies GmbH (Berlin, Germany). Guanidine hydrochloride, sodium hydroxide, dithiothreitol (DTT), ammonium hydrocarbonate, iodoacetamide, chloroform, methanol, MS-grade ACN, formic acid, sodium-EDTA, Trizma (Tris base), and all other chemicals and reagents were from Sigma (Saint Louis, MO). ProteaseMAX, sequencing-grade lysyl endopeptidase LysC, TPCK-treated trypsin, and urea were from Promega (Madison, WI). Solid phase extraction (SPE) cartridges were Strata-X, 33 μm, polymeric reversed phase (Part # 8B-S100-AAK) (10 mg/mL) purchased from Phenomenex (Torrance, CA).

**Table 2:** List of Peptides Used for CESs and AADAC in Multiple Species^a^

enzyme	peptides sequences	species (UniProt ID)
CES1	SYPIVNVSK*^392^	rabbit (P12337)
	FWANFAR*^505,503,468^	human (P23141), rat (Q63108), pig (F1RF16)
CES2	NIAHFGGNPGR*^194^	rabbit (G1T7P3)
	ADHGDELPFVFR*^466^	human (O00748)
	AGVHTFLGIPFAK*^67^	rat (A0A0G2K455)
CES3	LAFPEATEEE**K**^490^	human (Q6UWW8), rabbit (G1SNB1)
	ATGPETAQPEVDTALGR*^42^	rabbit (G1SNB1)
	TIASYTVDGTFFP**K**^322^	pig (I3LEI5)
	EATQPEVDTTLGR*^42^	
	NTIYPLTVDGTVFPK*^323^	human (Q6UWW8)
	TPEEILAEK*^338^	rat (P16303)
	FAPPQPAEPWNFVK*^78^	
AADAC	FWSEYFTTD**R**^258,258,257^	human (P22760), pig (A0A287AXR4), rat (Q9QZH8)
	YPGFLDVR*^318,318,317^	human (P22760), pig (A0A287AXR4), rabbit (Q7M370)
	TPTPGSLELAQK*^309^	rat (Q9QZH8)
	LDVVVVSTNY**R**^145^	rabbit (Q7M370)
Na^+^/K^+^ATPase	AAVPDAVGK*	all species

a The bold **K**/**R** indicate the ^13^C- and ^15^N-labeled residues required for quantitation. The number is the C-terminal residue of the peptide in the respective protein, or when the peptide is common for multiple species, the number is for the human isoform. SpikeTides_TQL peptides were used as internal standards. The C-terminal tetrapeptide tag on these is cleaved by the protease digestion. A peptide cocktail (20 nM each) used for simultaneous MRM quantitation was prepared in 0.1 M ammonium bicarbonate solution and ACN (1:4). Peptides marked with an asterisk (*) were used for protein quantitation. Note: the pig CES2 gene is absent from the reference genome [UCSC Genome Browser assembly ID: susScr11].

### Extraction and Homogenization of Ocular Tissues

Pig eyes were obtained from a local slaughterhouse (Lankamainen, Kuopio, Finland). The pig eyes were removed, cooled immediately on ice, and kept on ice during transportation to the laboratory. The delay from eye removal to receipt at the laboratory was maximally 2–3 h. Eyes from female Dutch-belted pigmented rabbits (Linköpings Kaninfarm, Sweden) and 9 week old Long-Evans male rats (Envigo, Netherlands) were prepared on site. To sacrifice the lab animals, a lethal dose of 60 mg/mL pentobarbital (Mebunat; Orion Pharma, Finland) was injected into the marginal ear vein of the animals. The studies with lab animals were approved by the local animal welfare committee (license # ESAVI/8621/04.10.07/2017).

Eyes were dissected essentially as before.^22^ Briefly, eyes were kept in ice-cold 1 × DPBS, pH 7.4. All preparations were performed on ice during the same day. All extraocular parts were removed, and the aqueous humor was aspirated with a 1 mL syringe 26 G needle. After this, all anterior tissues including conjunctiva, cornea, lens, and iris–ciliary body were separated. From the posterior segment, the vitreous and retina were collected and the RPE layer was scraped using a small brush into 1 mL of 1 × DPBS. Scraping from the eyecups was done twice to ensure complete collection of the RPE. The RPE suspensions from individual eyes were centrifuged at 6000*g* for 5 min at +4 °C, and the RPE pellets were pooled. Finally, the choroid and sclera were separated and cleaned from extraocular tissues. Specific tissues from four rabbit or six pig eyes constituted one individual tissue pool, and three such pools were prepared independently. Pooling helped to obtain sufficient material for both enzyme activities and proteomics studies and to decrease interindividual variation among outbred animals. All tissues were stored at −80 °C until the day of the homogenate preparation.

CES enzymes are localized in the endoplasmic reticulum,^2,45^ but some activity resides in the cytoplasm.^46^ Therefore, to prevent possible losses during sample preparation, we used whole tissue homogenates to characterize CES expression and activity in ocular tissues. The homogenization of pooled ocular tissues in 1 × DPBS buffer (3:1 v/w) was done with a Dounce homogenizer (Thomas Scientific; Swedesboro, NJ) with 5–10 strokes, depending on the ocular tissue. Aqueous humor and vitreous were homogenized without adding buffer. Tissue homogenization buffer was supplemented with protease inhibitor cocktail without phenylmethylsulfonyl fluoride (PMSF), a known inhibitor of esterases.^47,48^ After homogenization, all samples were sonicated at 750 W (Vibra-Cell VCX50 and a four-element microtip probe; Sonics & Materials Inc., Newton, CT) using four pulses of 45 s with a cooling interval of 15 s. After homogenization and sonication, all tissue homogenates were centrifuged at 10 000*g* for 15 min at +4 °C. Supernatants were collected, aliquoted, and stored at −80 °C as whole tissue homogenates. Homogenates for rat whole eye (covered by the license # ESAVI/8621/04.10.07/2017) and human liver, kindly donated by Prof. Olavi Pelkonen, from Oulu University Hospital,^44^ were also prepared as positive controls for the enzyme assays. Protein quantification of the homogenates was done using the Bradford assay (Bradford, 1976) and bovine serum albumin standard (0.25–2 mg/mL).

### Hydrolysis Assays

#### 4-Nitrophenol Acetate (NPA) Hydrolysis

NPA hydrolysis was performed with a slight modification to our previous protocol^22^ in clear flat-bottom 96-well Thermo Scientific Sterilin Microtiter plates at 37 °C. Diluted ocular samples (8 μg of protein in 80 μL of PBS) were pipetted into the reaction wells, and the background absorbance was measured at 405 nm for 10 min of preincubation. To start the reaction, an equal volume of NPA was added to a final concentration of 400 μM. Changes in absorbance at 405 nm were recorded with a Victor^2^ Microplate Reader (PerkinElmer Wallac 1420, St. Paul, MN) for 45 min with 90 s reading intervals. 4-Nitrophenol was used to generate a standard curve (5–400 μM).

#### d-Luciferin Methyl Ester (DME) Hydrolysis

The hydrolysis of DME was monitored with slight modifications using the published bioluminescence-based assay^13,21^ where recombinant firefly luciferase enzyme was used to detect the d-luciferin that formed in the reaction mixture. The DME hydrolysis reaction was done in white-coated 96-well plates (Thermo Scientific Sterilin Microtiter Plates). Tissue lysates (5 μg of protein) were preincubated in 0.1 M PBS, pH 6.5. The reaction was initiated by adding DME to a final concentration of 5 μM in a final volume of 200 μL. The hydrolysis of DME was allowed to proceed for 20 min at 37 °C. The formed d-luciferin was quantitated by an injection of 73 μL of the luciferase detection reagent [3 μg of rLuciferase, 50 nmol of ATP, and 50 nmol of MgCl_2_ in PBS], and the luminescence was measured immediately with a Victor^2^ Microplate Reader (560 nm, 1 s). Serial 2-fold dilutions of d-luciferin in 0.1 M PBS, pH 6.5 were used to generate the standard curve (0.156–10 μM).

#### Fluorescence Diacetate (FDA) Hydrolysis

A fluorometric assay was used to determine the hydrolysis of FDA to fluorescein.^17,49^ Diluted samples (5 μg of protein) were incubated at 37 °C in black 96-well Thermo Scientific Nunc F96 MicroWell plates. The background fluorescence was measured with a Victor^2^ Microplate Reader at excitation (485 nm) and emission (535 nm) for a 5 min preincubation at 37 °C. The reaction was started by adding FDA to a final 100 μM concentration to each well in a 200 μL total volume. Fluorescence readings were recorded every 60 s over 30 min. The fluorescein stock solution was used to generate a standard curve (0.156–10 μM) using 2-fold serial dilutions in 0.1 M PBS, pH 7.4.

The above assays were optimized for linearity to time and protein concentration in pilot studies, and the final organic solvent content was 0.5% (v/v) or less. Specific activities were calculated with the use of the standard curves, rate of the signal change at the linear response range, and the protein concentration. The reaction rates of blank samples (tissue sample replaced by the buffer, substrate replaced by solvent) were measured to control for any nonspecific hydrolysis of the probe substrates and subtracted from sample reaction rates. Three technical replicates were measured for each biological tissue pool, and the mean ± standard deviation (SD) was calculated.

### Quantification of Procaine and Phenacetin Metabolites by LC–MS

#### Procaine Hydrolysis

Procaine, a known human CES2 substrate,^2,17,50^ was incubated with rabbit and pig ocular tissues. After preincubation of the substrate (100 μM procaine in 0.1 M Tris-HCl, pH 7.4) at 37 °C, the reaction was initiated with tissue samples (20 μg of protein) in a total reaction volume of 200 μL. All the samples in parallel with the controls (no substrate or no enzyme) were incubated for 50 min at 37 °C on a shaker incubator (Heidolph Titramax 1000 platform shaker, Berlin Germany). The reaction was terminated by adding 200 μL of ice-cold ACN containing the internal standard procainamide (1 μM final concentration), followed by centrifugation at 4 °C for 10 min at 10 000*g*. Supernatants were collected for HPLC analysis. The standard curve was established using the hydrolysis product PABA with the concentration range 0.1–1000 nM.^51^

#### Phenacetin Hydrolysis

Phenacetin, a specific substrate for human AADAC,^18,19,24^ was incubated with ocular tissue homogenates. The substrate (4 mM, final concentration) was preincubated in the 0.1 M Tris-HCl, pH 7.4, buffer at 37 °C for 5 min, after which the tissue samples (20 μg of protein) were added to start the reaction in the total reaction volume of 200 μL. All the reaction and control samples were incubated for 50 min at 37 °C with shaking. The reaction was terminated by adding 200 μL of ice-cold ACN containing acetaminophen at an 1 μM final concentration as the internal standard, followed by centrifugation at 4 °C for 10 min at 10 000*g*. A standard curve using the metabolite *p*-phenetidine (0.1–100 μM)^24^ was established. The protein amount and incubation time were optimized from earlier pilot experiments for both procaine and phenacetin hydrolysis reactions. All incubations were performed in triplicate including the controls (control with no sample, control with no substrate), and the data are expressed as mean ± SD.

#### LC/Q Orbitrap MS Analysis

The metabolites from the above CES2 and AADAC assays were analyzed using a Waters (Milford, MA) Acquity ultra-performance liquid chromatographic (UPLC) coupled with a Q-Exactive Focus Orbitrap MS (Thermo Scientific). A Waters HSS T3 column (2.1 mm × 100 mm, 1.8 μm particle size) was used with elution gradients A (0.1% formic acid) and B (methanol) [0–0.5 min, 1% B; 0.5–4 min, 1%–20% B; 4–4.5 min, 20%–98% B; 4.5–5 min, 98% B; and 5–6 min, 98%–1% B]. The injection volume was 2 μL with a flow rate of 0.5 mL/min and a column temperature of 40 °C. A positive ionization mode with a capillary voltage of 3000 V and a mass range of 70–1000 *m*/*z* was used. The mass resolution was 35 000 (full width at half-maximum @ *m*/*z* 200) for a full scan and 17 500 for MS/MS in data-dependent acquisition mode. For desolvation and nebulizer, nitrogen gas (sheat gas 40, auxiliary gas 10, and sweep gas 3 arbitrary units) was used, the auxiliary gas temperature was 500 °C, and the capillary temperature was 320 °C. An external calibration system was used, and the data was processed using Thermo Xcalibur (version 4.1.31.9; Thermo Fisher Scientific Inc.).^53^

### Estimation of Kinetic and Inhibition Properties

Because of their high hydrolytic activity, corneal and retinal samples from pig and rabbit were used to determine the apparent kinetic parameters Michaelis–Menten constant (*K*_m_) and maximum reaction velocity (*V*_max_) for the hydrolysis of NPA, FDA, and DME. At least five different substrate concentrations within the following ranges (NPA 0.1–2 mM; FDA 3–100 μM; DME 0.3–10 μM) were used in triplicate using 5–8 μg of protein per well. The inhibition of the NPA, DME, and FDA hydrolytic activities by published esterase inhibitors (PMSF, digitonin, timolol, verapamil, and diltiazem)^47,54−56^ was conducted as described above with the following modifications. Inhibitors were tested at a final concentration range of 0.4–400 μM except for PMSF (0.3–2.5 mM). The substrate concentrations were reduced to apparent *K*_m_ values samples (NPA, 0.1 mM; DME, 5 μM; FDA, 10 μM). The control samples contained solvent instead of the inhibitor.

In all enzymatic measurements above, negligible hydrolysis rates were observed in the blank sample incubations, which indicated that nonenzymatic hydrolysis is minimal in the present experimental conditions (*data not shown*).

### Targeted Proteomics

#### *In Silico* Design of Peptides

Protein sequences were retrieved from the UniProt database (www.uniprot.org/help/uniprotkb). Unique peptide sequences for each protein were designed using BLAST^57^ and CLUSTALW algorithms.^58^ We aimed to use the peptide sequences that were also conserved across multiple species whenever possible. Peptides were selected according to peptide synthesis recommendations.^59^ The peptide for the membrane marker Na^+^/K^+^ ATPase was kindly provided by Dr. Mikko Gynther at UEF (Table 2).

#### Sample Preparation and Trypsin Digestion

The same samples used for enzymatic assays were used for targeted proteomic quantification. For the denaturation, tissue samples (50 μg of protein homogenate) were adjusted to 220 μL by adding solubilizer (7 M guanidine hydrochloride, 3 M Tris-HCl pH 8.5, and 0.5 M Na_2_-EDTA pH 8.0) in low-protein-binding tubes (Thermo Scientific). Then, an equal volume of DTT was added. The samples were mixed for 60 min at room temperature in the dark. Thereafter, a 2.5-fold excess of iodoacetamide was added and incubated for 60 min at room temperature in the dark to alkylate the sulfhydryl groups. For protein precipitation, the samples were cooled on ice and ice-cold methanol (600 μL), chloroform (150 μL), and deionized water (450 μL) were added sequentially with mixing by inversion. Tubes were centrifuged at 15 000*g* for 5 min at +4 °C. The upper layer containing lipids was discarded, and the samples were washed twice by ice-cold methanol (450 μL). The remaining protein pellets were resuspended by adding 6 M urea (9 μL) and 0.1 M Tris-HCl (36.5 μL) and mixing for 10 min at room temperature.

To ensure complete resuspension, intermittent sonication was done (30 s per cycle: Branson 2510) while keeping samples on ice. For the protein digestion, 0.5 μg of LysC (1/100-fold of protein amount) and proteaseMax (final concentration 0.05%) were added to the samples processed above. Subsequently, 0.15 pmol of heavy-labeled peptides (Table 2) were added to each 50 μg protein sample tube and incubated for 3 h at 37 °C.

Thereafter, TPCK-treated trypsin (0.5 μg) was added to each tube and incubated overnight at 37 °C. The digestion was terminated by adding 3 μL of 20% formic acid per sample, followed by centrifugation at 15 000*g* at +4 °C. The supernatants were transferred to fresh vials, and an aliquot corresponding to 22.7 μg of original protein homogenate from each digest was taken for targeted quantitative proteomic analysis. The digests were treated with SPE (C18, 10 mg/mL cartridges) in preparation for nanoLC–MS/MS analysis. Cartridges were conditioned with 250 μL of methanol followed by 250 μL of deionized water. A sample was added, and the cartridges were washed with 150 μL of deionized water. Peptides were eluted with 250 μL of ACN/0.1% formic acid (60/40) into 0.5 mL LoBind Eppendorf tubes. Samples were then evaporated to dryness and reconstituted in 50 μL of 2% ACN. The samples were centrifuged at 13 400*g* for 5 min, and the supernatants were transferred to deactivated vial inserts for analysis.

### Targeted Quantitative Analysis with NanoLC–MS/MS

The analysis was performed as previously described^60,61^ on a nanoAcquity (Waters) coupled to a QTRAP 5500 with a NanoSpray III source (SCIEX, Framingham, MA). The system control was with nanoAcquity UPLC Console software (Waters) and Analyst 1.5 (SCIEX). The mobile phase was as follows: A, 1% ACN and 0.1% formic acid in deionized water and B, 100% ACN. The injection volume was 0.2 μL corresponding to 0.091 μg of sample or 0.4% of the nominal sample amount (22.7 μg). A sample was loaded onto a Symmetry C18 trap column (Waters, part # 186006527, 5 μm particle size, 180 μm × 20 mm). The trapping flow was 15 μL/min of mobile phase A for 1 min. The analytical column was a BEH130 C18, 1.7 μm particle size, 150 mm × 100 mm (Waters, part # 186003550). The flow rate was 1.3 μL/min, and the gradient is shown in Table S1, with the total run time being 35 min. The mass spectrometer was operated in the positive mode with the ion spray voltage set at 4000. A 90 s scheduled acquisition window was used for each peptide. For a selection of multiple reaction monitoring (MRM) transitions to be used (two labeled and two unlabeled per peptide), a range of transitions were first predicted by Skyline software (version 2.6, University of Washington). The five transitions giving the highest response for SIL peptide standards were selected, and the collision energies were optimized by repeated injection onto the system (declustering potentials were left unchanged). The two MRMs giving the best response were then selected for use in concentration calculations. MRM data was processed using MultiQuant 2.0.2 (SCIEX). Peptide concentrations were calculated using area ratios of endogenous (unlabeled) to a known amount of SIL peptide standard (0.15 pmol to the initial 50 μg of protein). Equality of response between the SIL and unlabeled peptides was assumed. One peptide, generally that giving the highest value, was used to report the concentration of the relevant protein with a second peptide, if available, being used as confirmatory.

### Statistical Analysis

The differences among hydrolytic activities were analyzed by ANOVA using the SigmaPlot software (Sigma Plot 13.0, Systat Software Inc., San Jose, CA), followed by Mann–Whitney U test for statistical comparisons between species and between tissues. Between species, statistically significant differences are ranked by the asterisk signs (in the order of *, *p* < 0.05; **, *p* < 0.01, and *** *p* < 0.001). Between tissues of the same species, statistically significant differences in comparison to conjunctiva are represented by the dagger sign (†, *p* < 0.05). Nonlinear regression analysis to determine the *K*_m_, *V*_max_, and IC_50_ values were done using GraphPad Prism (GraphPad, 5.04 Software Inc., San Diego, CA). For IC_50_, logarithms of inhibitor concentrations were plotted against the relative response (0 μM inhibitor = 100%).

## Results

### NPA, DME, and FDA Hydrolytic Activities

**Figure 1:**
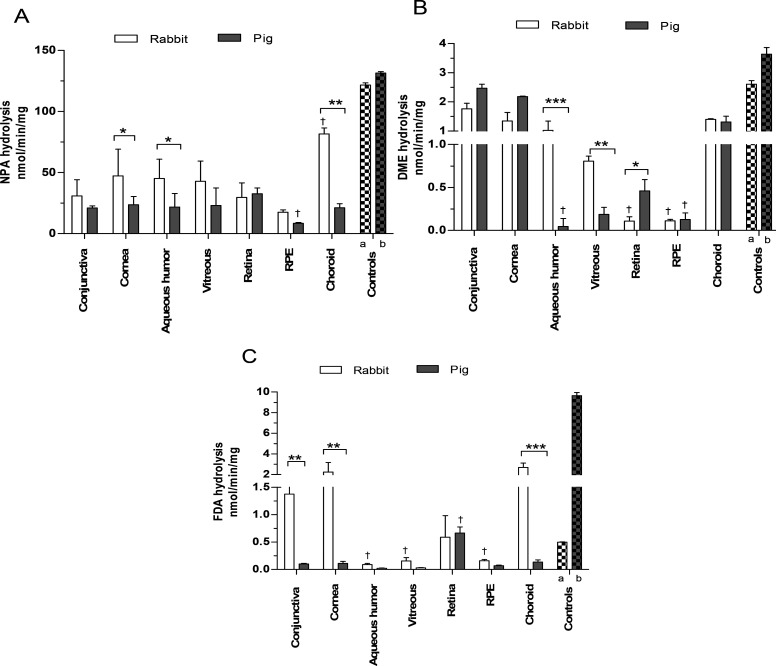
Hydrolysis of (A) NPA, (B) DME, and (C) FDA in ocular tissue samples from pigmented rabbits (*white columns*), pig (*gray columns*), and positive controls (*patterned columns*; a = rat whole eye homogenate, b = human liver homogenate). The data shown are mean ± SD from three different pools, each measured in three technical replicates. Statistical comparisons between species (*) and between tissues in the same species as compared to conjunctiva (†) were done by the Mann–Whitney nonparametric test. Statistically significant differences are ranked as follows: *†, *p* < 0.05, **, *p* < 0.01, ***p < 0.001.

NPA is often used to measure the activities of various hydrolytic enzymes including CESs, AADAC, acetylcholinesterase, and arylesterase in multiple species.^2,22,23,62^ The positive controls, rat whole eye and human liver homogenates, showed high rates of NPA hydrolysis (Figure 1A) as expected from the literature.^22,40,62^ Rabbit samples showed quite similar rates of 30–45 nmol/min/mg protein for NPA hydrolysis among most tissue homogenates, while the lowest activity was observed in RPE (17.5 ± 2.7 nmol/min/mg) and the highest in the choroid (81.6 ± 6.8 nmol/min/mg). For the pig, NPA hydrolysis rates were very similar, around 20 nmol/min/mg among all tissues, except the lowest rate was observed again with RPE (8.4 ± 1.0 nmol/min/mg).

Overall, the enzymatic activities in pig tissues tended to be lower than those in the rabbit, corresponding to earlier data,^22^ and statistically significant species differences were noted for the cornea, aqueous humor, and choroid.

DME has been suggested as a highly selective and sensitive substrate for detecting CES1 activity in the human liver.^13^ To date, there are no reports on DME hydrolytic activities in ocular tissues or in other rabbit or porcine tissues. Control samples showed the hydrolysis rates for human liver and rat whole eye homogenate at 3.6 ± 0.7 and 2.6 ± 0.2 nmol/min/mg, respectively (Figure 1A). In rabbit ocular tissues, hydrolysis rates were high (≥1 nmol/min/mg) in the conjunctiva, cornea, and choroid, moderate (0.5–1.0 nmol/min/mg) in the aqueous humor and vitreous, and low (0.1–0.5 nmol/min/mg) in the RPE and retina (Figure 1B). Similarly, in pig, DME hydrolysis was high (≥1 nmol/min/mg) in the conjunctiva, cornea, and choroid, more moderate (about 0.5–1 nmol/min/mg) in the retina, and relatively low (0.1–0.2 nmol/min/mg) in the RPE, aqueous humor, and vitreous (Figure 1B). Statistically significant species differences were noted for aqueous humor and vitreous, which had about 5–10-fold higher activity in the rabbit than in the pig.

FDA has been indicated as a probe substrate for the determination of CES2 activity in human liver^17,49^ while its hydrolysis has not been studied so far in ocular tissues or in rabbit or porcine tissues. Rabbit tissues showed (Figure 1C) high hydrolysis rates (>1.0 nmol/min/mg) in the choroid, cornea, and conjunctiva as compared to other tissues (retina, 0.5–1 nmol/min/mg; RPE, aqueous humor and vitreous, 0.1–0.2 nmol/min/mg). FDA hydrolysis was remarkably low in most pig ocular tissues (<0.1 nmol/min/mg) except retinal samples (0.6 ± 0.1 nmol/min/mg). Much higher hydrolysis rates (up to 20-fold) were seen in most rabbit ocular tissues as compared to porcine tissues (Figure 1C), and cornea, choroid, and conjunctiva showed statistically significant species differences.

### Procaine and Phenacetin Hydrolysis

**Figure 2:**
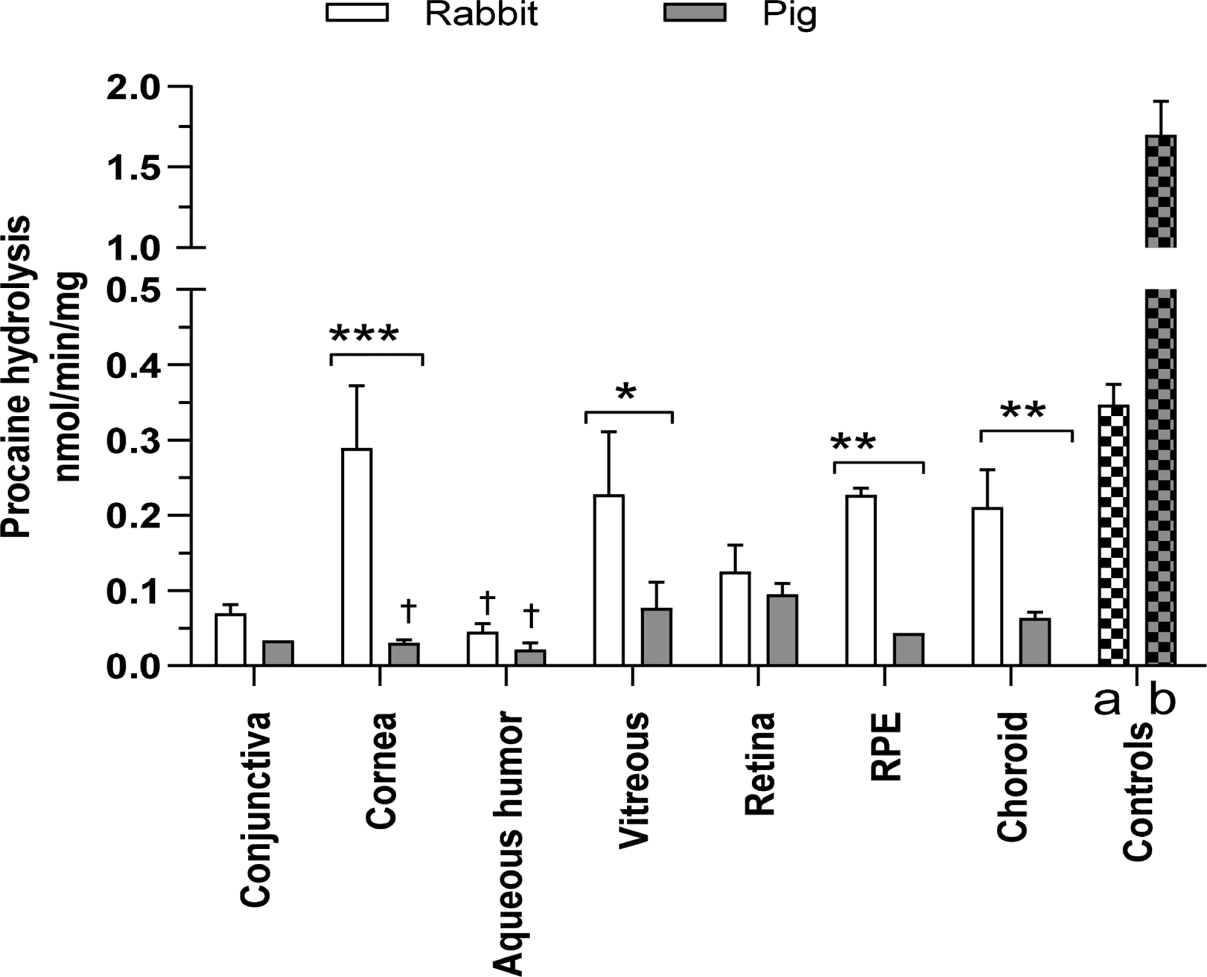
Hydrolysis of procaine to PABA in ocular tissues (conjunctiva, cornea, aqueous humor, vitreous, retina, RPE, and choroid) of rabbit (light gray) and pig (dark gray). The data shown are mean ± SD from three different pools, each measured in three technical replicates. Statistical comparisons between species (*) and between tissues in the same species as compared to vitreous (†) were done by Mann–Whitney nonparametric test. Statistically significant differences are ranked as follows: *, *p* < 0.05, **, *p* < 0.01, ***p < 0.001.

The presence of CES2 in ocular tissues was also probed by measuring the formation of human CES2-selective metabolite PABA from procaine.^50,51^ Rabbit ocular tissues showed PABA formation in all tissues (Figure 2). The activities were rather similar in the cornea, vitreous, RPE, and choroid (∼0.2–0.3 nmol/min/mg) while the aqueous humor and conjunctiva displayed 4–7-fold lower levels (∼0.04–0.1 nmol/min/mg). In contrast, the pig ocular tissues showed more evenly distributed procaine hydrolysis rates in all tissues, ranging from the lowest in aqueous humor (0.02 nmol/min/mg) to the highest in the retina (0.09 nmol/min/mg). Rabbit tissues tended to contain higher rates of PABA formation as compared to pig tissues, similarly to FDA hydrolysis.

Phenacetin is hydrolyzed to *p*-phenetidine by the human AADAC.^24^ However, the levels of *p*-phenetidine formed in the incubation from the ocular tissue of rabbit and pig were below the detection limit (∼0.1 pmol/min/mg) in the present experimental conditions (*data not shown*). The positive control samples showed significant activities as expected.

### Kinetics and Inhibition Studies

Kinetic parameters for NPA, DME, and FDA hydrolysis were determined in pig and rabbit corneal and retinal tissues and compared to the existing literature (Table 3 and Figure 1). For all three substrates, the observed *K*_m_ values were within 4-fold or less of the reported values employing different human enzyme sources (Table 3). Unfortunately, there is only limited published data on pig liver CES1 and rabbit corneal CESs with NPA. Given the overall similarity of the *K*_m_ values across different species and tissues, we judged that they were sufficiently accurate to select substrate concentrations for the subsequent inhibition studies.

**Table 3:** Kinetics for CES Mediated Hydrolysis Reactions

substrate	main enzyme	*K*_m_ (μM) literature data	references	*K*_m_(μM) (this study)
NPA	multiple esterases	198 ± 17 (HLM)^a^	(23, 32, 49, and 63)	125 ± 13 in pig cornea
		68 ± 15 (rabbit cornea)		263.2 ± 15 in rabbit cornea
		520 ± 60 (pig liver CES1)		
DME	CES1	≈5 (rhCES1)	(13)	2.7 ± 1.3 in pig cornea
				2.5 ± 0.8 in rabbit cornea
FDA	CES2	4.87 ± 0.51 (HLM)	(16, 49, and 64)	16 ± 1.6 in pig retina
		28.8 (HLM)		5.5 ± 0.4 in rabbit cornea

a HLM, human liver microsomes; rhCES, recombinant human CES1 or CES2.

Inhibition studies were conducted first with the general esterase substrate NPA to validate the hydrolysis assays (Table 4). PMSF, a generic hydrolase inhibitor, inhibited NPA hydrolysis in pig cornea rather gradually to the maximum of about 80% at the highest concentration. A unique IC_50_ value could not be determined, but a 50% inhibition was observed at about 200 μM (Figure 2). Under the present experimental conditions, timolol and diltiazem could not appreciably inhibit NPA hydrolysis (maximal inhibition of 10% and 20% at the highest 400 μM concentration). The human CES1-selective inhibitor digitonin showed significant inhibition of NPA hydrolysis (∼40%) and a quantifiable IC_50_ value (38 ± 3 μM).

**Table 4:** Inhibition for CES Mediated Hydrolysis Reactions^a^

inhibitor	main human enzyme	IC_50_	*K*_i_	references	IC_50_ or extent of inhibition (this study)
PMSF	most esterases	0.541 mM	0.56 mM	(47, 48, and 54)	NPA: ∼200 μM in pig cornea
digitonin	CES1	9.2 ± 0.4 μM (rhCES1)	NA	(54 and 55)	NPA: 38 ± 3 μM in pig cornea
		25.8 μM (rhCES1)			DME: >200 μM in pig cornea, ∼30% at 200 μM in rabbit cornea, <20% in both conjunctivas
timolol	CES2	20% inhibition at 200 μM	NA	(2, 54, and 55)	NPA: no inhibition in pig cornea
					FDA: <20% inhibition in rabbit retina, no inhibition in pig retina
verapamil	CES2 > CES1	7.94 μM (rhCES2)	11.5 ± 1.20 μM (HLM)	(20, 54, and 65)	NPA: 28 ± 7 μM in pig cornea;
			3.84 ± 0.99 μM (rhCES2)		FDA: 38 ± 8 μM in rabbit retina, no inhibition in pig retina
diltiazem	CES2 > CES1	3.98 μM (rhCES2)	2.89 ± 0.39 μM (HLM)	(20, 54, and 65)	NPA: no inhibition in pig cornea
		0.25 ± 0.02 μM (rhCES2)			

a HLM, human liver microsomes; NA, not available; rhCES, recombinant human CES1 or CES2. Inhibition of substrate hydrolysis was done in pig cornea for NPA, in pig and rabbit cornea and conjunctiva for DME, and in pig and rabbit retina for FDA.

Digitonin was also used to inhibit DME hydrolysis in pig and rabbit tissues with high DME activity. In rabbit conjunctiva, digitonin showed no inhibition in DME hydrolysis, while a slight 15% inhibition was observed in pig conjunctiva (Figure 3A,B). In pig and rabbit cornea, digitonin showed slightly stronger maximal inhibitions of 45% and 30%, respectively (Figure 3C,D). Rabbit and pig retina that showed relatively high FDA activities were used to inhibit FDA hydrolysis using a human CES2-selective inhibitor verapamil and timolol. No inhibition of FDA hydrolysis was seen in pig retina for either inhibitor (Figure 4A,B). However, about 40% inhibition of FDA hydrolysis was evident in rabbit retina with verapamil but less with timolol (Figure 4C,D). These data suggest that pig and rabbit CES enzymes are somewhat affected by human CES inhibitors, but the less than complete inhibition suggests that multiple enzymes participate in the hydrolysis of these substrates.

### Quantification of Protein Expression

Considering the reference samples, the human liver homogenate displayed a higher expression of CES1 (64 ± 8 pmol/mg) as compared to that of CES2 (30.6 ± 2.1 pmol/mg), as expected from a recent report,^66^ while the expression of CES3 was very low (∼0.2 pmol/mg). We report here for the first time that the expression of CES1 (∼3.0 pmol/mg) and CES3 (∼2.3 pmol/mg) proteins was higher than CES2 levels (∼0.2 pmol/mg) in rat whole eye. In rabbit ocular samples, a relatively low and even expression of CES1 (∼0.2 pmol/mg) was seen in all tissues except conjunctiva and choroid. CES2 was present in rabbit conjunctiva, retina, and choroid (0.3, 0.2, and 0.1 pmol/mg, respectively), while other tissue levels were below the lower limit of quantitation (LLOQ, 0.1 pmol/mg protein).

Pig cornea showed the highest expression of CES1 (8.0 ± 2.5 pmol/mg), while the expression was clearly detectable in choroid and conjunctiva (∼0.7 pmol/mg), followed by vitreous (∼0.2 pmol/mg), and all other pig tissues were below the LLOQ. CES2, in accordance with its absence in the pig genome (UCSC Genome Browser assembly ID: susScr11), was not measured in the pig tissues. Finally, CES3 showed no or very low expression in rabbit or pig tissues, respectively, and AADAC seemed to be absent in the ocular tissues of both species (Table 5) in contrast to their presence in the reference samples.^57^ Thus, there were clear species differences in the expression of CES1 and CES2. CES1 was expressed at higher levels in pig tissues as compared to the rabbit, apart from retina and RPE where the situation was the opposite. CES2 was found in the rabbit ocular tissues and is not believed to be present in pig due to the absent *CES2* gene.

**Table 5:** Concentrations of CESs and AADAC Proteins Determined in Rabbit and Pig Ocular Tissues^a^

		ocular tissues (pmol/mg protein)							reference samples (pmol/mg protein)	
enzyme	species	conjunctiva	cornea	aqueous humor	vitreous	retina	RPE	choroid	rat whole eye	human liver
CES1	rabbit	<LLOD	0.14 ± 0.05	0.1 ± 0.03	0.2 ± 0.01	0.2 ± 0.03	0.1 ± 0.02	<LLOD	3.0 ± 0.1	64 ± 8.0
	pig	0.7 ± 0.1	8.05 ± 2.5	<LLOD	0.2 ± 0.01	<LLOQ*	<LLOD	0.7 ± 0.2		
CES2	rabbit	0.3 ± 0.1	<LLOQ*	<LLOD	<LLOD	0.2 ± 0.1	<LLOD	0.1 ± 0.07	0.2 ± 0.05	30.6 ± 2.1
	pig	ND	ND	ND	ND	ND	ND	ND		
CES3	rabbit	<LLOD	<LLOD	<LLOD	<LLOD	<LLOD	<LLOD	<LLOD	2.3 ± 0.1	0.2 ± 0.09
	pig	<LLOD	0.1 ± 0.01	<LLOD	<LLOD	<LLOD	<LLOQ*	<LLOD		
AADAC	rabbit	<LLOD	<LLOD	<LLOD	<LLOD	<LLOD	<LLOD	<LLOD	0.3 ± 0.01	0.8 ± 0.2
	pig	<LLOD	<LLOD	<LLOD	<LLOD	<LLOD	<LLOD	<LLOD		
Na^+^/K^+^ ATPase	rabbit	1.5 ± 0.3	1.4 ± 0.7	<LLOD	1.1 ± 0.3	10.3 ± 1.3	6.0 ± 2.5	4.5 ± 1.3	1.5 ± 0.3	2.7 ± 0.2
	pig	1.3 ± 0.1	1.4 ± 0.2	<LLOD	0.3 ± 0.1	17 ± 7.5	7.0 ± 1.7	4.0 ± 1.2		

a LLOD, lower limit of detection = 3 × signal/noise ratio; LLOQ, lower limit of quantification = 0.1 pmol/mg protein; * protein detectable but not quantifiable; ND, not done, due to absence of the *CES2* gene. The protein expression levels of CES1, CES2, CES3, and AADAC were determined in the rabbit and pig ocular tissues. Na^+^/K^+^ ATPase served as a marker for the membrane fraction. Each data point calculated in pmol/mg presented above is the average of three pools ± SD.

The Na^+^/K^+^ ATPase is a membrane-bound marker^59,67^ that was used to indicate the enrichment of membrane fraction in the samples. In both rabbit and pig ocular tissues, retina and RPE exhibited abundant expression (6–17 pmol/mg), followed by choroid (∼4.5 pmol/mg), cornea, and conjunctiva (∼1.3 pmol/mg). The relatively cell-free aqueous humor and vitreous had no or little Na^+^/K^+^ ATPase expression as expected. This suggests a similar efficiency of protein extraction between all samples.

### Correlation between the Enzyme Activity and Protein Expression

Rabbit ocular tissues showed no significant correlation between the human CES1-related DME activity and the quantity of CES1 protein (Figure 5A). In addition, the selective human CES1-selective inhibitor digitonin attenuated DME hydrolysis only by 30% in rabbit cornea or less in conjunctiva (Table 4 and Figure 3). On the contrary, a significant positive correlation (*r*^2^ = 0.803) was observed between DME activity and CES1 content in pig ocular tissues (Figure 5B). We also observed that the extent of DME inhibition by digitonin in pig conjunctiva and cornea was stronger than that in the rabbit (Table 4 and Figure 3) and correlated with CES1 contents in these pig tissues.

No correlation was observed between the FDA activity and CES2 quantity in rabbit ocular tissues (Figure 5C), although the human CES2-selective inhibitor verapamil reduced FDA hydrolysis significantly in rabbit retina (Table 4 and Figure 4), the tissue with highest CES1 content. For the pig, the *CES2* gene is absent and thus no correlation could be established. In line with the absence of CES2, neither verapamil or another human CES2 inhibitor timolol blocked FDA activity in pig retina, indicating that another hydrolase is responsible for this activity. However, FDA and procaine hydrolysis, often used to determine human CES2-dependent activity, correlated strongly (*r*^2^ = 0.810) in pig ocular tissues but poorly in rabbit tissues (Figure 4D,E). Finally, the emerging esterase AADAC was included in proteomics and enzymatic assays; the results indicated negligible expression and activity of AADAC in ocular tissues of both species.

## Discussion

Ocular drug metabolism is still poorly defined. Knowledge on the presence and levels of drug-metabolizing enzymes in ocular tissues would be essential to aid in ophthalmic drug development and delivery. We aimed to generate data on the drug-metabolizing capacity of hydrolyzing enzymes in ocular tissues of rabbits and pigs, which are commonly employed preclinical species for ocular drug development. Numerous ocular drugs or prodrugs containing an ester or amide bond are liable to hydrolysis in ocular tissues of various species.^33,38,62,68^ Although esterase activity is well-presented, the detailed activity and expression data on CES isoforms in the eye is still ambiguous.^22,40^ For the first time in ocular tissues, we adopted substrates and inhibitors that have been used to characterize human CES^13,16,25^ and AADAC^18,21^ activities, because the respective rabbit and pig enzymes have not been expressed *in vitro* and characterized for their substrate specificity. To our knowledge, there are no earlier reports on the quantitative protein expression of these enzymes in rabbit and pig ocular tissues. The accumulation of such information will help design experiments in ocular drug metabolism and aid in the interpretation of resulting data. Detailed knowledge of esterase expression will assist in evaluating the feasibility of an esterase-dependent prodrug approach, the design of esterase-cleavable drug conjugates for ocular drug therapy, and the extent of ocular effects of systemically administered drugs that are substrates for hydrolytic enzymes. The species differences in ocular drug hydrolysis also affect the design and interpretation of translational and toxicity studies.

The hydrolysis of the general hydrolase substrate, NPA, has been detected in ocular tissues of multiple species including pig and rabbit.^22,40,62^ In a recent study^22^ from our laboratory, most of the pig and albino rabbit ocular tissues displayed very similar rates of NPA hydrolysis as what we report here. The only exceptions were pig aqueous humor and rabbit choroid, with slightly higher (30%) specific activities in the present study. These modest differences are explained by our fully optimized reaction conditions and a pigmented rabbit strain used here. A similar study was earlier conducted with a naphthyl prodrug to determine esterase hydrolysis in the anterior eye segment. The relative hydrolysis rates of naphthyl prodrugs were similar to those of our study (cornea > conjunctiva > aqueous humor). Again, pigmented rabbits showed 10%–100% higher esterase activity as compared to albino animals.^8,40,62^

In addition to this, dipivalyl epinephrine, an ester prodrug showed a twice as large hydrolysis rate in the corneal epithelium of pigmented rabbit as that seen in albinos.^33^ A ganciclovir ester prodrug was hydrolyzed in descending order of reaction rate in choroid, retina, and vitreous homogenates, a pattern similar to that of NPA hydrolysis in our study.^37,38^ Similarly, loteprednol etabonate was hydrolyzed more rapidly in rabbit cornea than in aqueous humor.^39^ Likewise, topically applied latanoprost, an ester prodrug, showed complete hydrolysis in rabbit cornea.^34^ In summary, across all studies, the cornea appeared to have the highest and the vitreous and aqueous humor had the lowest hydrolytic activities for different ester substrates. However, the isoforms responsible for these reactions remain unknown.

To the best of our knowledge, scant literature is available on the inhibition of ocular carboxylesterases. Lee reported 30%–70% inhibition of naphthyl acetate hydrolysis in rabbit cornea, iris–ciliary body, and aqueous humor by several cholinesterase inhibitors at 1000 μM while *p*-chloromercuribenzoate and EDTA, which are inhibitors of carboxylesterase and arylesterase, respectively, blocked the hydrolysis only by 10%–30%.^62^ Our inhibition analysis showed that the generic esterase inhibitor PMSF blocked the NPA hydrolysis by more than 80% in pig cornea. Although a unique IC_50_ value could not be determined, 50% inhibition was reached at ∼200 μM, which is rather close to the reported IC_50_ value of ∼500 μM in other tissues.^47,48^ The selective human CES1 inhibitor digitonin^54^ inhibited NPA hydrolysis in pig cornea by about 40%, while timolol or diltiazem had little if any effect. Verapamil has been reported to inhibit CES2^20,54^ or both CES1 and CES2,^65^ and it significantly blocked pig corneal NPA hydrolysis. Although additional enzymes likely contributed to the hydrolysis of NPA, these findings seem consistent with the higher expression of CES1 enzyme in pig cornea that leads to substantial CES1 inhibition by digitonin and verapamil. The lack of inhibition by timolol or diltiazem is in line with negligible CES2 expression in pig cornea.

The human CES1-selective substrate DME^13^ was hydrolyzed at the highest rates in rabbit and pig conjunctiva, cornea, and choroid. However, there was a poor correlation between the DME activity and CES1 content in rabbit tissues. On the contrary, the extent of DME inhibition by digitonin was greater (30%) in rabbit cornea that expressed the CES1 protein than in conjunctiva lacking CES1. In contrast, DME activity and CES1 content correlated in pig. This finding is well supported by the inhibition of DME hydrolysis, where digitonin showed a stronger inhibitory effect in pig than in rabbit tissues. The rates of human CES2-selective FDA hydrolysis were the highest in the rabbit conjunctiva, cornea, and choroid. Again, no correlation between FDA hydrolysis and CES2 content was found in rabbit tissues. This hypothesis was supported by the fact that no inhibition was shown in FDA hydrolysis by verapamil. Poor correlations between hydrolysis rates of DME or FDA substrates and CES1 or CES2 contents, respectively, and less than complete blockade by inhibitors both suggest that other hydrolytic enzymes in the rabbit ocular tissues contribute to the metabolism of these human-selective substrates.

FDA has been suggested as a human CES2-specific substrate,^17,49^ but later studies revealed that it is also hydrolyzed by human AADAC.^14,16^ Subsequently, we determined the hydrolysis of procaine, a more specific substrate for human CES2,^50^ and of phenacetin for AADAC.^19,24^ Rabbit ocular tissues showed a much higher activity for FDA and procaine than the pig, which is in line with the lack of CES2 in the latter species, while both AADAC content and phenacetin hydrolysis were undetectable in both species’ tissues.

Recently, a global proteomics technique has revealed proteins expressed in individual human ocular tissues^42^ although not quantitatively. Various human eye proteome studies have detected CES1 in cornea, vitreous, RPE/choroid, and retina but not in aqueous humor.^42^ Our quantitative data lists all of these tissues as CES1-positive with the exception of pig retina and pig aqueous humor (Table 4). Other global proteomic studies conducted in pig or rabbit ocular tissues^69,70^ have only detected the presence of CES2 in the rabbit aqueous humor.^71^ This contrasts with our studies where we could quantify CES2 in rabbit conjunctiva, rabbit retina, and rabbit choroid (Table 5).

Because we could not devise common peptide probes for pig and rabbit enzymes, a caveat remains that a comparison of absolute protein contents between these species may be biased due to the differential properties of the peptides. However, the use of exact quantities of heavy-labeled peptides ensures their coelution and identical fragmentation with the endogenous unlabeled peptide from the sample, thus allowing determination of the protein concentration, and dilution experiments have shown excellent linearity of the response. Therefore, an earlier analysis of CES1 and CES2 in other species^72^ using separate peptides showed only few-fold differences. Another counterargument can be made on the basis of the following: the inhibition of DME hydrolysis by CES1-preferring digitonin was much more efficient in pig than in rabbit tissues, and its inhibition in conjunctiva was less than that in cornea. Both findings match well with the rank order of the observed CES1 levels. Similarly, the genetically known lack of CES2 expression in the pig was reflected in a negligible inhibition of FDA hydrolysis in pig retina by verapamil, but a clear reduction of this activity was in rabbit retina. Although not conclusive, these explanations suggest that our comparative analysis of protein expression was not grossly distorted by the use of separate peptide probes.

Collectively, the poor correlation between CES levels and marker activities and the modest extent of DME or FDA inhibition both indicate that the expression and substrate profiles of CES1 and CES2 differ across species. These differences should be taken into consideration in follow-up studies. We cannot yet exclude participation by other hydrolyzing enzymes such as paraoxonases^73^ for the marker activities we utilized. Conclusions that are more definitive require heterologous expression of these rabbit and porcine CES isoforms and characterization of their preferred substrates and inhibitors in the future. Nevertheless, this study generated fundamental knowledge about hydrolytic activities in the eye and added to the literature on ocular metabolism in preclinical species. The present study also employed, for the first time, a quantitative proteomics approach to ocular tissues. Due to the low level of proteins detected, future studies should aim to improve the detection limit and address methods to enrich samples to enhance enzyme quantitation.

## Conclusions

Our study is the first to assess the metabolism of multiple substrates and quantitative protein expression of carboxylesterases in rabbit and pig ocular tissues. Significant tissue and species differences in these parameters were found: CES1 was present in all rabbit tissues except conjunctiva and choroid, while CES2 was expressed in these two tissues and retina. Pig appeared to express CES1 in most ocular tissues and CES3 at low levels in the cornea, while AADAC was absent in both species. The enzymatic and inhibition studies suggest that, in rabbit and pig ocular tissues, additional esterases participate in the hydrolysis of NPA, DME, FDA, and procaine, with a greater contribution to DME by CES1. Our findings will advance the understanding of the ocular drug metabolism and its application to ocular drug delivery and prodrug pharmacokinetics.

## Supporting Information Available

Tables of chromatography gradient program data and MRM parameters for the analysis of CESs and AADAC peptides and figures of Michaelis–Menten kinetics of NPA, DME, and FDA hydrolysis, inhibition of NPA hydrolysis in pig cornea using general and human isoform-selective inhibitors, inhibition of DME hydrolysis by digitonin, inhibition of FDA hydrolysis by human CES2-selective inhibitors, and correlation between hydrolytic activities and CES isozyme contents in pig and rabbit ocular tissues (PDF)

## Abbreviations

AADAC: arylacetamide deacetylase
ACN: acetonitrile
ATP: adenosine triphosphate
BSA: bovine serum albumin
CES: carboxylesterase
DPBS: Dulbecco’s phosphate-buffered saline
DME: d-luciferin methyl ester
DMSO: dimethyl sulfoxide
DTT: dithiothreitol
FDA: fluorescein diacetate
LC: liquid chromatography
LLOQ: lower limit of quantitation
LLOD: lower limit of detection
MRM: multiple reaction monitoring
MS: mass spectrometry
NPA: 4-nitrophenol acetate
PABA: *p*-aminobenzoic acid
PBS: phosphate-buffered saline
PMSF: phenylmethylsulfonyl fluoride
rh: recombinant human
RPE: retinal pigment epithelium
SIL: stable isotope labeled
SPE: solid phase extraction
