# Supplementary material for: Carboxylesterase Activities and Protein Expression in Rabbit and Pig Ocular Tissues
Source: Mol Pharm. 2021 Feb 17;18(3):1305–16. doi: 10.1021/acs.molpharmaceut.0c01154 (PMC8023712; doi:10.1021/acs.molpharmaceut.0c01154)
Supplement: Supplementary file 1 — mp0c01154_si_001.pdf [file mp0c01154_si_001.pdf]

SUPPLEMENTARY MATERIAL to

Carboxylesterase activities and protein expression in rabbit and pig ocular tissues

Anam Hammid[1], John K. Fallon[2], Toni Lassila[3], Giulia Salluce[4], Philip C. Smith[2], Ari Tolonen[3], Achim Sauer[5], Arto Urtti[1,6,7], Paavo Honkakoski[1,8*]

[1]School of Pharmacy, University of Eastern Finland, Yliopistonranta 1 C, 70210 Kuopio, Finland
[2]Division of Pharmacoengineering and Molecular Pharmaceutics, Eshelman School of Pharmacy, University of North Carolina at Chapel Hill, Campus Box 7355, Chapel Hill, NC 27599-7355, USA
[3]Admescope Ltd, Typpitie 1, 90620 Oulu, Finland
[4]Centro Singular de Investigación en Química Biolóxica e Materiais Moleculares (CiQUS), Departamento de Química Orgánica, Universidade de Santiago de Compostela, 15782 Santiago de Compostela, Spain
[5]Department of Drug Discovery Sciences, Boehringer Ingelheim Pharma GmbH & Co. KG, 88397 Biberach, Germany
[6]Institute of Chemistry, Saint Petersburg State University, Universitetskii pr. 26, 198584 Saint Petersburg, Russia
[7]Faculty of Pharmacy, University of Helsinki, Viikinkaari 5 E, 00790 Helsinki, Finland
[8]Division of Pharmacotherapy and Experimental Therapeutics, Eshelman School of Pharmacy, University of North Carolina at Chapel Hill, Campus Box 7569, Chapel Hill, NC 27599-7569, USA

*Corresponding Author: paavo.honkakoski@uef.fi; Tel.: +358 40 355 2490

CONTENTS

Supplementary Table S1. The chromatography gradient program for analysis of CESs and AADAC

Supplementary Table S2. MRM parameters for the analysis of CESs and AADAC peptides

Supplementary Figure S1. Michaelis-Menten kinetics of NPA & DME (pig & rabbit cornea) and FDA (pig retina & rabbit cornea) hydrolysis

Supplementary Figure S2. Inhibition of NPA hydrolysis in pig cornea using general and human isoform-selective inhibitors

Supplementary Figure S3. Inhibition of DME hydrolysis by digitonin, a human CES1-selective inhibitor

Supplementary Figure S4. Inhibition of FDA hydrolysis by human CES2-selective inhibitors

Supplementary Figure S5. Correlation between hydrolytic activities and CES isozyme contents in pig and rabbit ocular tissues

Supplementary Table S1. The chromatography gradient program for analysis of the CES and AADAC peptides (flow rate was 1.3 µL/min; mobile phase A, deionized water containing 1 % acetonitrile and 0.1 % formic acid; mobile phase B, acetonitrile).

	Time (min)	Mobile phase A (%)	Mobile phase B (%)
**1**	0	100	0
**2**	24	58	42
**3**	24.5	5	95
**4**	27.5	5	95
**5**	28	100	0
**6**	35	100	0

Supplementary Table S2. MRM parameters for the analysis of CESs and AADAC peptides. Three MRMs, labeled and unlabeled, are shown for each peptide but only the first two were used for the concentration determinations. MRMs are listed roughly in decreasing order of intensity.

Enzyme	Peptide sequence	CE (V)	Hydrophobicity (From SkylineCalc)	RT (min)	Precursor ion (m/z)	Product ion (m/z) (y or b)
**Na^+/K^+**	AAVPDAVGK	18.8	10.62	9.5	414.23	586.32 (y6)
**ATPase**	(light)	15.8			414.23	242.15 (b3)
		22.8			414.23	293.66 (y6+2)
	AAVPDAVG**K**	18.8			418.25	594.34 (y6)
	(heavy)	15.8			418.25	242.15 (b3)
		22.8			418.25	297.68 (y6+2)
**CES1**	SYPIVNVSK	23	20.68	12.2	503.78	378.73 (y7+2)
	(light)	22			503.78	756.46 (y7)
		30			503.78	546.32 (y5)
	SYPIVNVS**K**	23			507.79	382.75 (y7+2)
	(heavy)	22			507.79	764.49 (y7)
		30			507.79	554.35 (y5)
**CES1**	FWANFAR	23.3	27.42	15.3	456.23	578.30 (y5)
	(light)	23.3			456.23	507.27 (y4)
		24.3			456.23	764.38 (y6)
	FWANFA**R**	23.3			461.23	588.31 (y5)
	(heavy)	23.3			461.23	517.28 (y4)
		24.3			461.23	774.39 (y6)
**CES2**	NIAHFGGNPGR	14.3	15.84	10.0	380.53	456.73 (y9+2)
	(light)	20.3			380.53	329.19 (y3)
		20.3			380.53	704.35 (y7)
	NIAHFGGNPG**R**	14.3			383.86	461.73 (y9+2)
	(heavy)	20.3			383.86	339.20 (y3)
		20.3			383.86	714.36 (y7)
**CES2**	ADHGDELPFVF	17.1	31.09	16.7	468.23	333.19 (y5+2)
	R	18.1			468.23	738.31 (b7)
	(light)	22.1			468.23	665.38 (y5)
		17.1			471.57	338.20 (y5+2)
	ADHGDELPFVF	18.1			471.57	738.31 (b7)
	**R**	22.1			471.57	675.39 (y5)
	(heavy)					
**CES2**	AGVHTFLGIPF	19.2	36.1	17.4	453.26	462.27 (y4)
	AK	19.2			453.26	231.64 (y4+2)
	(light)	19.2			453.26	783.41 (b8)
		19.2			455.93	470.30 (y4)
	AGVHTFLGIPF	19.2			455.93	235.65 (y4+2)
	A**K**	19.2			455.93	783.41 (b8)
	(heavy)					
**CES3**	LAFPEATEEEK	19.5	20.58	10.9	421.87	575.77 (y10+2)
	(light)	24.5			421.87	559.26 (b10+2)
		28.5			421.87	430.21 (b8+2)
	LAFPEATEEE**K**	19.5			424.55	579.78 (y10+2)
	(heavy)	24.5			424.55	559.26 (b10+2)
		28.5			424.55	430.21 (b8+2)

**CES3**	NTIYPLTVDGT	37.8	36.04	17.7	832.95	1173.65 (y11)
	VFPK	28.8			832.95	492.25 (b4)
	(light)	24.8			555.63	763.40 (y7)
		37.8			836.96	1181.68 (y11)
	NTIYPLTVDGT	28.8			836.96	492.25 (b4)
	VFP**K**	24.8			558.31	771.42 (y7)
	(heavy)					
**CES3**	TPEEILAEK	27.4	17.74	12.0	515.28	464.75 (y8+2)
	(light)	27.4			515.28	831.45 (y7)
		30.4			515.28	702.40 (y6)
	TPEEILAE**K**	27.4			519.29	468.77 (y8+2)
	(heavy)	27.4			519.29	839.47 (y7)
		30.4			519.29	710.43 (y6)
**CES3**	ATGPETAQPEV	20.7	22.22	12.8	571.62	479.25 (y9+2)
	DTALGR (light)	28.7			571.62	632.34 (y6)
		30.7			571.62	741.36 (b15+2)
	ATGPETAQPEV	20.7			574.96	484.26 (y9+2)
	DTALG**R** (heavy)	28.7			574.96	642.34 (y6)
		30.7			574.96	741.36 (b15+2)
**CES3**	FAPPQPAEPWN	36.2	33.14	17.4	814.42	705.36 (y12+2)
	FVK	21.1			543.28	395.72 (y6+2)
	(light)	21.1			543.28	790.42 (y6)
		36.2			818.43	709.38 (y12+2)
	FAPPQPAEPWN	21.1			545.96	399.73 (y6+2)
	FV**K**	21.1			545.96	798.45 (y6)
	(heavy)					
**CES3**	TIASYTVDGTFF	31.7	32.75	15.9	773.89	851.41 (b8)
	PK	20.7			516.26	851.41 (b8)
	(light)	20.7			516.26	736.39 (b7)
	TIASYTVDGTFF	31.7			777.91	851.41 (b8)
	P**K**	20.7			518.94	851.41 (b8)
	(heavy)	20.7			518.94	736.39 (b7)
**CES3**	EATQPEVDTTL	34.4	18.49	11.6	708.85	987.51 (y9)
	GR	29.4				
	(light)	33.4			708.85	460.19 (b4)
		34.4			708.85	494.26 (y9+2)
	EATQPEVDTTL	29.4			713.86	997.52 (y9)
	G**R**	33.4				
	(heavy)				713.86	430.19 (b4)
					713.86	499.26 (y9+2)
**AADAC**	FWSEYFTTDR	30.2	32.45	16.2	676.30	1018.45 (y8)
	(light)	30.2			676.30	802.37 (y6)
		32.2			676.30	931.42 (y7)
	FWSEYFTTD**R**	30.2			681.31	1028.46 (y8)
	(heavy)	30.2			681.31	812.38 (y6)
		32.2			681.31	941.42 (y7)
**AADAC**	YPGFLDVR	24.3	28.07	15.7	483.76	402.22 (y7+2)
	(light)	27.3			483.76	706.34 (y6)
		20.3			483.76	693.32 (b6)
	YPGFLDV**R**	24.3			488.76	407.23 (y7+2)
	(heavy)	27.3			488.76	716.40 (y6)
		20.3			488.76	693.32 (b6)

**AADAC**	TPTPGSLELAQ K (light)	32.2	21.72	12.3	621.34	942.53 (y9)
		34.2			621.34	471.77 (y9+2)
		33.2			621.34	300.16 (b3)
	TPTPGSLELAQ **K** (heavy)	32.2			625.35	950.55 (y9)
		34.2			625.35	475.78 (y9+2)
		33.2			625.35	300.17 (b3)
**AADAC**	LDVVVVSTNYR (light)	30.6	25.89	14.4	632.85	739.37 (y6)
		30.6			632.85	838.44 (y7)
		29.6			632.85	640.30 (y5)
	LDVVVVSTNY **R** (heavy)	30.6			637.85	749.38 (y6)
		30.6			637.85	848.45 (y7)
		29.6			637.85	650.31 (y5)

***CE,** collision energy; **RT**, retention time

Supplementary Figure S1.

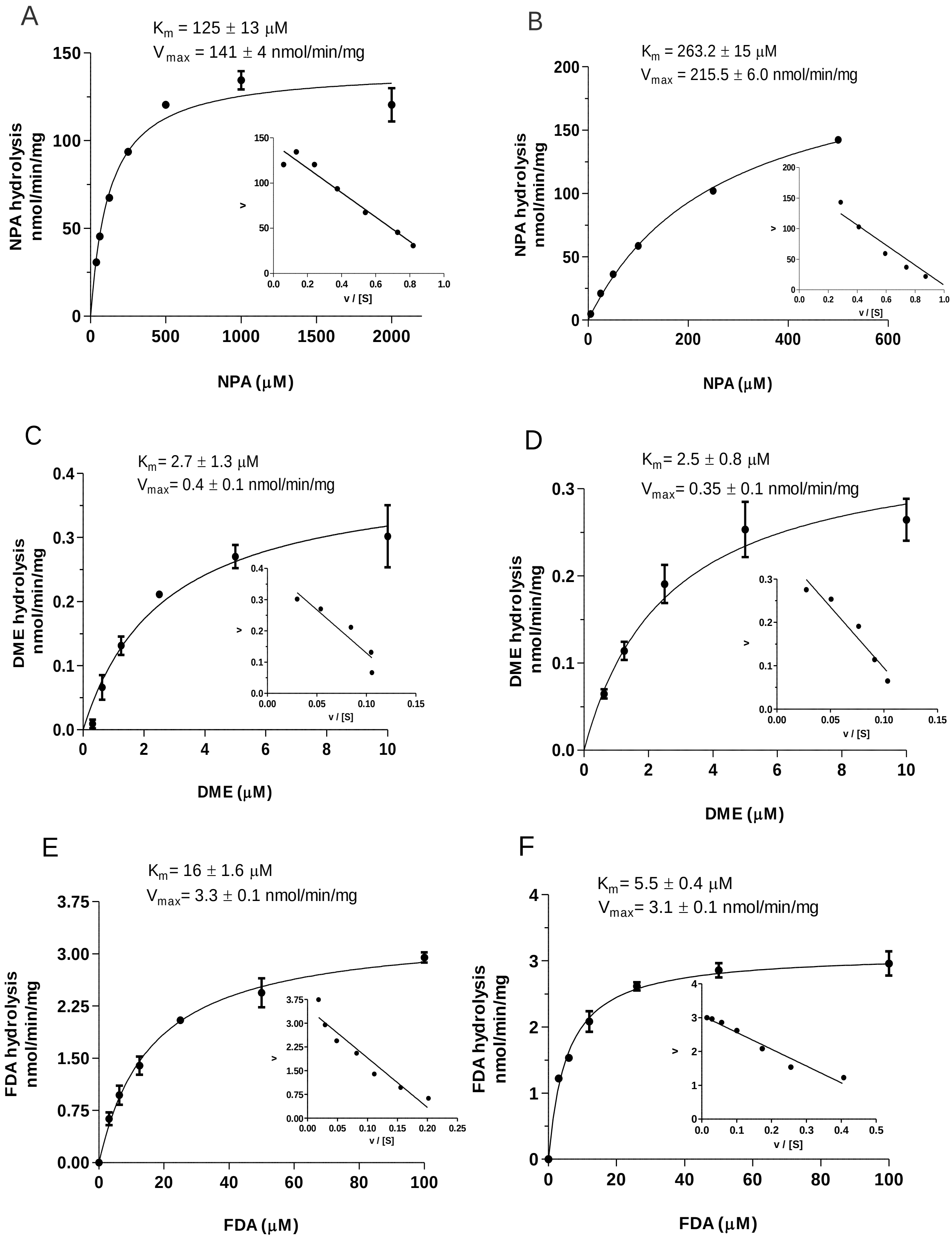

Figure S1. Michaelis-Menten kinetics of NPA & DME (pig & rabbit cornea) and FDA (pig retina & rabbit cornea) hydrolysis. The reactions were measured in the presence of increasing concentrations of (A & B) NPA, (C & D) DME, and (E & F) FDA substrates.

The data shown are mean ± SD of three replicates for each data point. The apparent V_{max} and K_m values were calculated using non-linear regression in GraphPad Prism, and the inset shows the Eadie-Hofstee plot of the data.

Supplementary Figure S2.

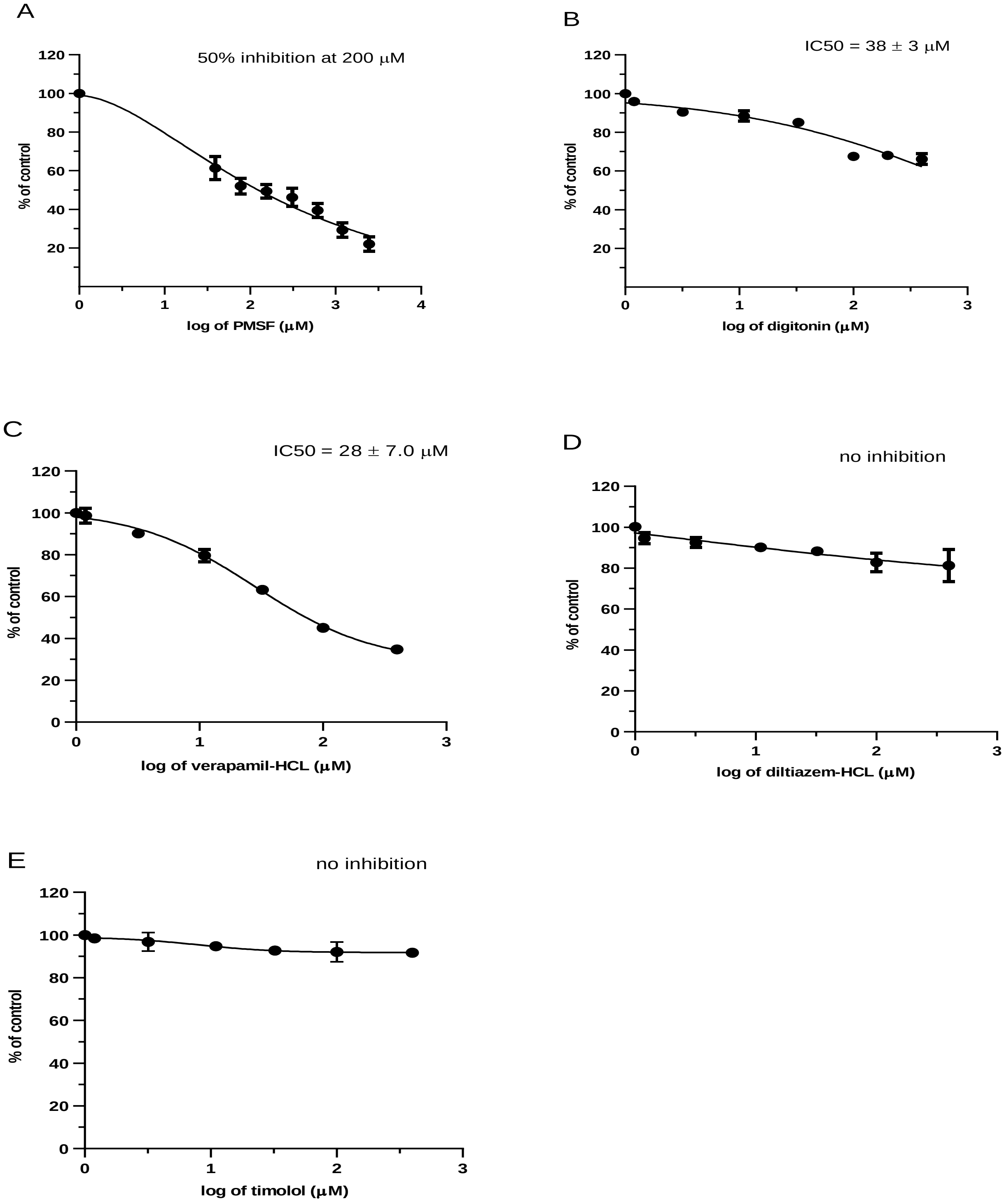

Figure S2. Inhibition of NPA hydrolysis in pig cornea using general and human isoform-selective inhibitors. Hydrolysis assays were conducted at 0.1 mM NPA with increasing concentrations of PMSF (A), digitonin (B), verapamil (C), diltiazem (D) and timolol maleate (E). The activity in the presence of DMSO solvent control was set at 100%. The IC_{50} values were calculated using non-linear regression analysis. Each data point represents the mean ± SD of three replicates.

Supplementary Figure S3.

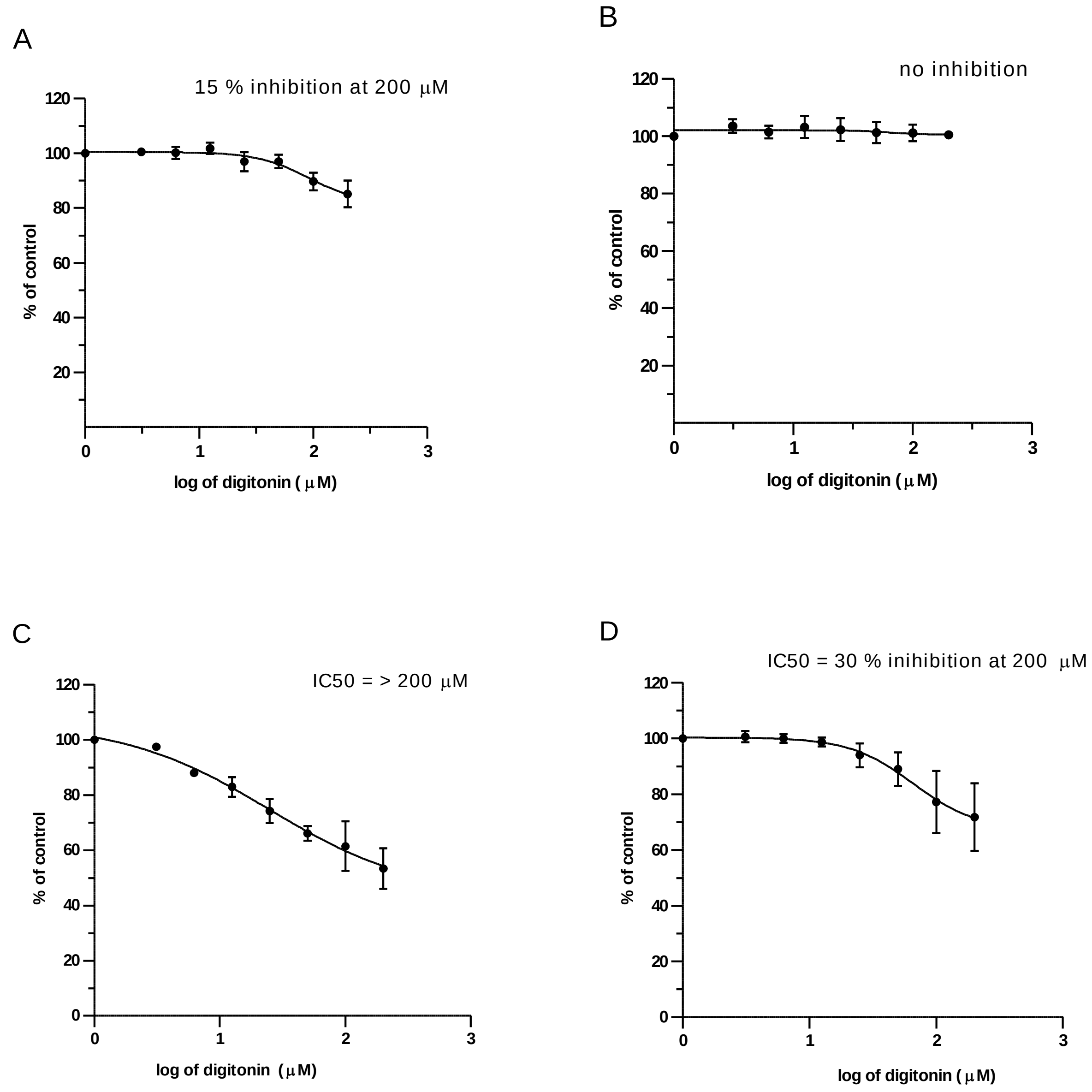

Figure S3. DME hydrolysis by digitonin, a human CES1-selective inhibitor. Hydrolysis assays were conducted at 5 µM DME with increasing concentrations of inhibitor using pig and rabbit conjunctiva (A & B), and cornea (C & D), respectively. The IC50 values were calculated using non-linear regression analysis. Each data point represents the mean ± SD of three replicates.

Supplementary Figure S4.

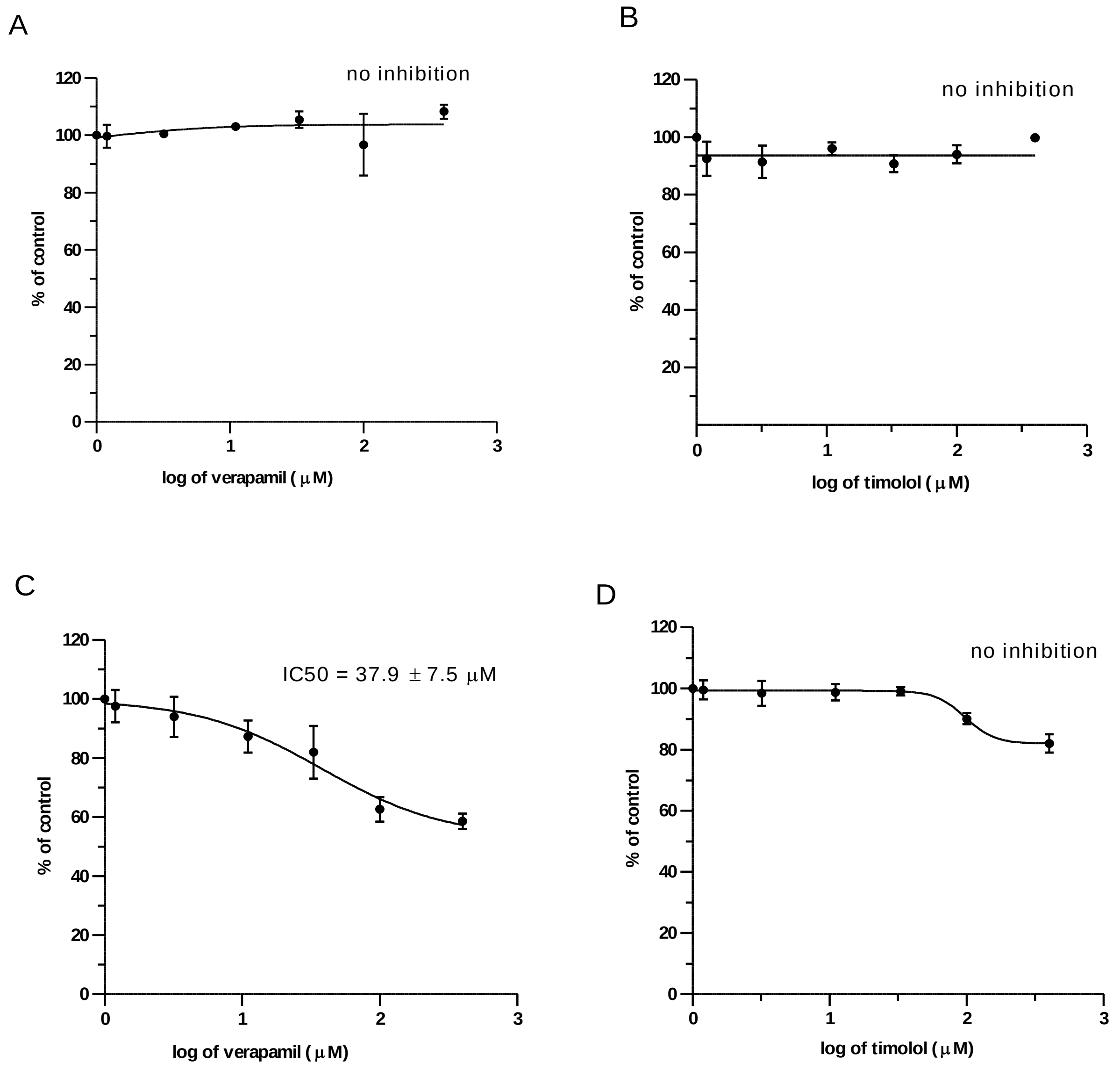

Figure S4. Inhibition of FDA hydrolysis by human CES2-selective inhibitors. Hydrolysis assays were conducted at 10 µM FDA with increasing concentrations of verapamil using pig and rabbit retina (A, C), and timolol using pig and rabbit retina (B, D). The activity in the presence of DMSO solvent control was set at 100%. The IC_{50} values were calculated using non-linear regression analysis. Each data point represents the mean ± SD of three replicate

Supplementary Figure S5.

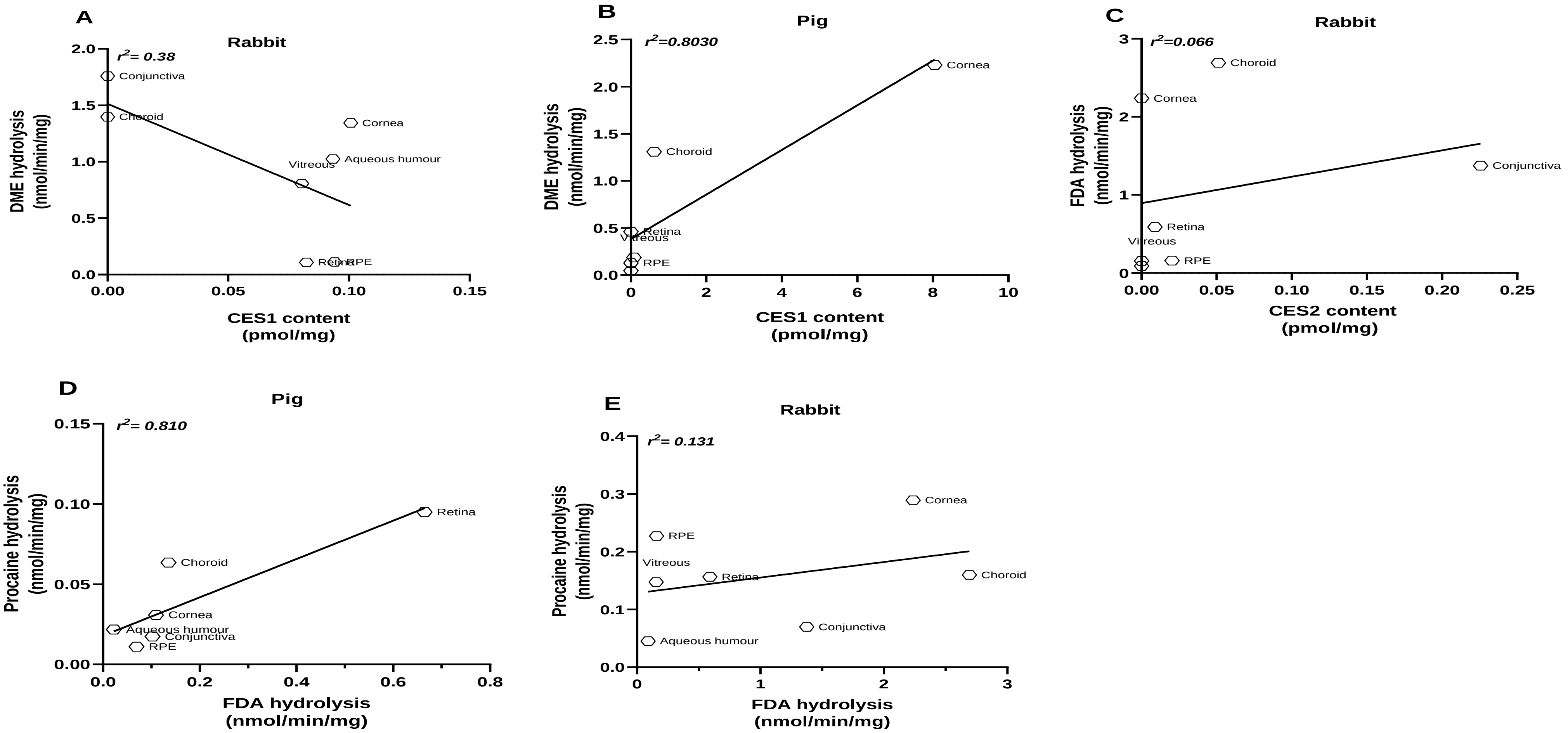

Figure S5. Correlation between hydrolytic activities and CES isozyme contents in pig and rabbit ocular tissues. Correlation between DME hydrolysis and expression level of CES1 in rabbit (A) and pig (B) ocular tissues, and between FDA hydrolysis and rabbit CES2 content (C) are shown. The correlation between procaine and FDA hydrolytic activities of pig (D) and rabbit (D) tissues are shown.
